# Engineering tissue morphogenesis: taking it up a Notch

**DOI:** 10.1016/j.tibtech.2022.01.007

**Published:** 2022-02-15

**Authors:** Laura A. Tiemeijer, Sami Sanlidag, Carlijn V.C. Bouten, Cecilia M. Sahlgren

**Affiliations:** 1 Faculty for Science and Engineering, Biosciences, Ábo Akademi University, Turku, Finland; 2 Department of Biomedical Engineering, Eindhoven University of Technology, Eindhoven, The Netherlands; 3 Institute for Complex Molecular Systems (ICMS), Eindhoven University of Technology, Eindhoven, The Netherlands; 4 InFLAMES Research Flagship Center, Ábo Akademi University, Turku, Finland; 5 Turku Bioscience Centre, Ábo Akademi University and University of Turku, Turku, Finland

## Abstract

Recreating functional tissues through bioengineering strategies requires steering of complex cell fate decisions. Notch, a juxtacrine signaling pathway, regulates cell fate and controls cellular organization with local precision. The engineering-friendly characteristics of the Notch pathway provide handles for engineering tissue patterning and morphogenesis. We discuss the physiological significance and mechanisms of Notch signaling with an emphasis on its potential use for engineering complex tissues. We highlight the current state of the art of Notch activation and provide a view on the design aspects, opportunities, and challenges in modulating Notch for tissue-engineering strategies. We propose that finely tuned control of Notch contributes to the generation of tissues with accurate form and functionality.

## Engineering shape and form through Notch signaling

Current bioengineered grafts generally provide living replacements for relatively simple tissues. However, they are not yet able to recreate complex tissue architectures and functionalities. Tissue architecture can be copied, for example by bioprinting, but recreating complex tissue function requires in-depth understanding of the developmental processes of their native counterparts as well as approaches to re-engineer and control these processes. Tissue development comprises spatiotemporal coordination of cells, and this coordination is crucial for the acquisition of cellular patterns during tissue development and regeneration [1,2]. Therefore, facilitation of intercellular coordination via the delivery of spatially controlled signals is crucial for engineering patterning and morphogenesis.

**Figure 1:**
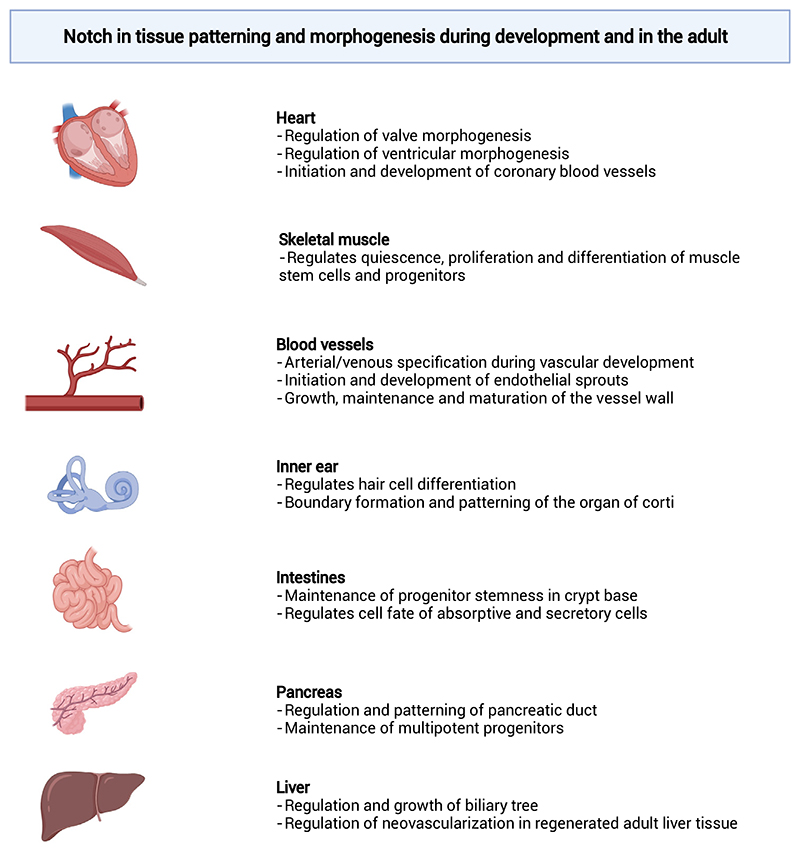
Selected examples of Notch signaling in tissue patterning and morphogenesis. Notch is involved inthe tissue patterning and morphogenesis of multiple organs and tissues, both during development and in adult tissue patterning. Examples include (but are not restricted to) heart, skeletal muscle, vascular system, inner ear, intestine, pancreas, and liver.

Juxtacrine cell signaling guides cell fate, patterning, and boundary formation through direct contacts between neighboring cells and offers handles to control cell signaling with local precision [2–4]. Notch is a conserved regulator of cell fate decisions, and the juxtacrine Notch signaling pathway orchestrates numerous fundamental morphogenic processes [5,6]. Upon interactions between a Notch receptor presented on one cell and a ligand presented on another cell, the receptor releases an active intracellular fragment, which then moves to the nucleus where it regulates cell intrinsic developmental gene networks. Notch also controls multicellular patterning via lateral induction (see Glossary) and lateral inhibition. These signaling modes contribute to tissue organization by promoting uniform or differential cell fate decisions between adjacent cells, respectively [7] (see Figure I in Box 1). In this way, Notch dictates form and function during tissue morphogenesis (Figure 1).

**Figure 2:**
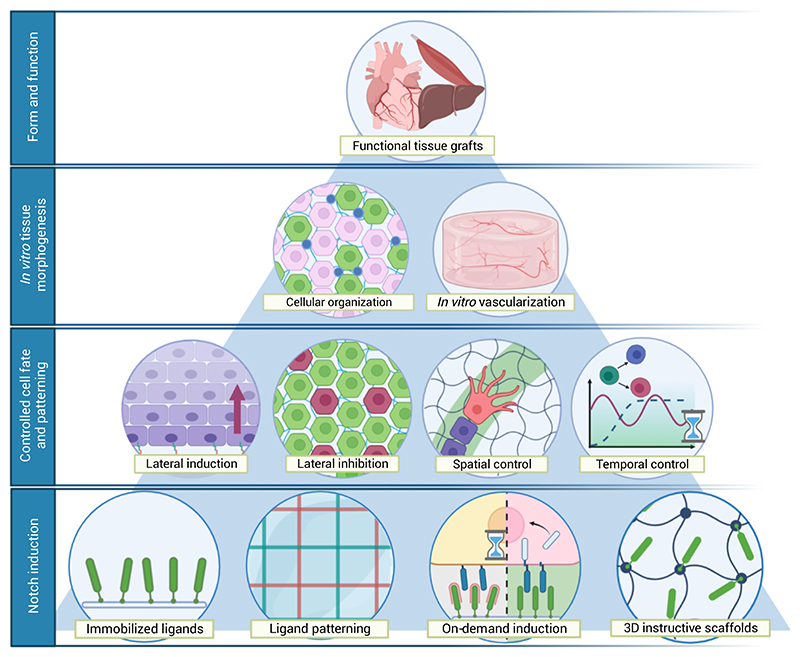
Notch signaling offers a handle for creating functional tissue grafts with correct form and function. Notch controls the acquisition of tissue architecture as well as the propagation of the vascular network within the tissue during development and in the adult. This patterning can be mimicked in artificial microenvironments through spatiotemporal control of lateral induction and inhibition events via modulation of Notch to create tissues with accurate form and function. This can be achieved via immobilized ligands and appropriate material design.

Owing to its engineering-friendly characteristics – as outlined in the following text – Notch emerges as a potent lever for controlling cell fate decisions and patterning in tissue engineering applications. Although Notch signaling has been used to control cell differentiation and expansion *in vitro* and *in vivo* (Table 1), attention has only recently been drawn to its use for guiding tissue patterning. This has been spurred by the development of engineering toolboxes and material design concepts (Figure 2, Key figure). In this review we discuss the role of Notch in tissue patterning and the rationale for targeting it for tissue engineering purposes. We provide an outlook on the current state of material-mediated bioengineering approaches for activating and fine-tuning Notch, as well as technical considerations and design challenges in its use for patterning tissues.

## Notch in morphogenesis and native tissue patterning

The Notch pathway regulates developmental genetic programs, and there is extensive crosstalk between the Notch pathway and many other signaling pathways involved in organ development [8–10]. Notch thus acts as a gatekeeper for the molecular cascades that lead to important cell fate decisions during tissue morphogenesis [6,11–13]. Examples include the formation of heart valves, bile ducts in the liver, and pancreatic tissues, the regeneration of intestinal crypts and skeletal muscle, and tissue vascularization (Figure 1) [6,11,13–16]. Modulation of Notch thus allows control over cell fate in engineered environments. Notch can guide tissue patterning in multiple tissue-engineering contexts and aid in culturing organ and tissue mimics for biomedical research in the laboratory (Figure 2, first tier). Although the role of Notch signaling in various biological processes has previously been the subject of excellent reviews [6,7], in this section we highlight some examples, focusing on the potential of Notch for tissue patterning in the context of tissue engineering.

### Notch controls tissue patterning during development

Notch guides cell-intrinsic processes that are important for morphogenesis, such as proliferation, migration, and differentiation, and also defines tissue fields and boundaries via lateral patterning mechanisms. Notch is involved in cell fate decisions that give rise to functional compartments in the developing heart. During the morphogenesis of valves, Notch mediates the epithelial-to-mesenchymal transition predominantly through delta-like ligand (Dll) 4 activity to promote the formation of the endocardial cushions. Later, endocardial Jagged 1 (Jag1) mediates cushion fusion by restricting mesenchymal proliferation [17]. During ventricular development, endocardial Dll4 activity in the trabecular base and subsequent myocardial Jag1/2 activity control trabeculae formation and myocardial compaction, respectively [18]. The same ligands guide the formation of coronary vessels throughout the compacting myocardium [19]. In some tissues, lateral patterning events are prominent, and they significantly guide tissue shape and function. For example, during aortic arch development, Jag1 promotes the differentiation of vascular smooth muscle cells (VSMCs) from progenitors through lateral induction [20]. In the developing pancreas, Dll1-mediated lateral inhibition regulates progenitor maintenance and differentiation as well as pancreatic epithelium branching via tip versus trunk cell selection [14]. During inner-ear development, the organ of Corti is patterned through consecutive lateral induction and inhibition events mediated by Jag1/2 and Dll1, which draw the tissue boundaries and also direct sensory hair versus supporting cell differentiation [7,21]. All in all, Notch controls important cell fate decisions which are important for gain of form and function during development, and can also mediate morphogenesis across tissue planes through lateral patterning events. Thus, controlling Notch may offer a handle to control morphogenesis in a wide range of tissues and organs.

### Notch drives tissue patterning in adults

The role of Notch in tissue patterning during development also often translates to their regeneration in adults. Notch contributes to the regeneration and homeostasis of many tissues and organs including skeletal muscle, vascular wall, heart, liver, and intestines in the adult, for example by regulating the balance between the stem cell pool and the differentiated cells, as well as regulating multiple differentiation events and cellular organization [11–13,16,22]. For example, in the intestinal crypts, lateral inhibition between intestinal stem cells (ISCs) and their neighbors maintains high Notch activity in the ISCs and favors their expansion at the crypt base. A similar lateral inhibition mechanism controls the balance between the adsorptive and the secretory cells in the gastrointestinal tract [13]. In another example, Notch regulates the remodeling of adult arteries by regulating VSMC plasticity through lateral induction [22,23]. Furthermore, Notch modulation has been shown to stimulate stem cell proliferation and differentiation *in vitro* and *in vivo* [24–26]. For example, activation of Notch signaling via immobilized Dll1 has aided in the *ex vivo* expansion of human cord blood progenitor cells [24,27]. In addition, Notch signaling via Jag1 overexpression aids regeneration of pressure-overloaded adult mouse heart [28]. Taken together, these examples suggest that Notch signaling is a potent target for *in situ* as well as *in vitro* re-engineering of adult tissues for regeneration.

### Notch guides vascular network formation and angiogenesis

Vascularization is imperative for recreating functional tissue grafts because larger engineered tissue constructs cannot rely on diffusion of oxygen nutrients and waste owing to the diffusion limit of 200 –m [29]. Because Notch is a crucial regulator of angiogenesis – the formation of new blood vessels from existing vessels – it may be developed as a tool for *de novo* tissue vascularization [30] (Figure 2, second tier). Notch ligands Dll4 and Jag1 antagonistically guide the branching and propagation of the vascular tree, in cooperation with vascular endothelial growth factor receptor (VEGFR) signaling [31]. More specifically, Dll4-mediated lateral inhibition regulates endothelial cell specification into tip and stalk cells, whereas Jag1 induces a tip cell phenotype by modulating Dll4/Notch1 signaling. In addition, during vessel maturation, Jag1-mediated Notch activation controls vascular maturation by recruiting VSMCs and pericytes [20,32,33]. In the adult artery, Notch signaling regulates vascular wall thickness and mechanical homeostasis, as well as VSMC contractility [22,23]. These make Notch modulation a potent approach for guiding vascularization of engineered tissues, as well as for engineering functional contractile arteries.

## Notch: an engineering-friendly tool for tissue patterning

Absolute control over cell fate in complex multicellular tissues is difficult to achieve, but the use of a strong molecular trigger such as Notch to steer cell fate decisions may prove very valuable for tissue morphogenesis and maturation. In addition to its physiological significance in tissue patterning, the Notch signaling pathway possesses properties which make it an engineeringfriendly tool. Functionalization of materials, such as 2D substrates, scaffolds, or particles, with Notch ligands has many advantages, as discussed in the following text.

### Notch signaling is dose-dependent and ligand-specific

**Figure 3:**
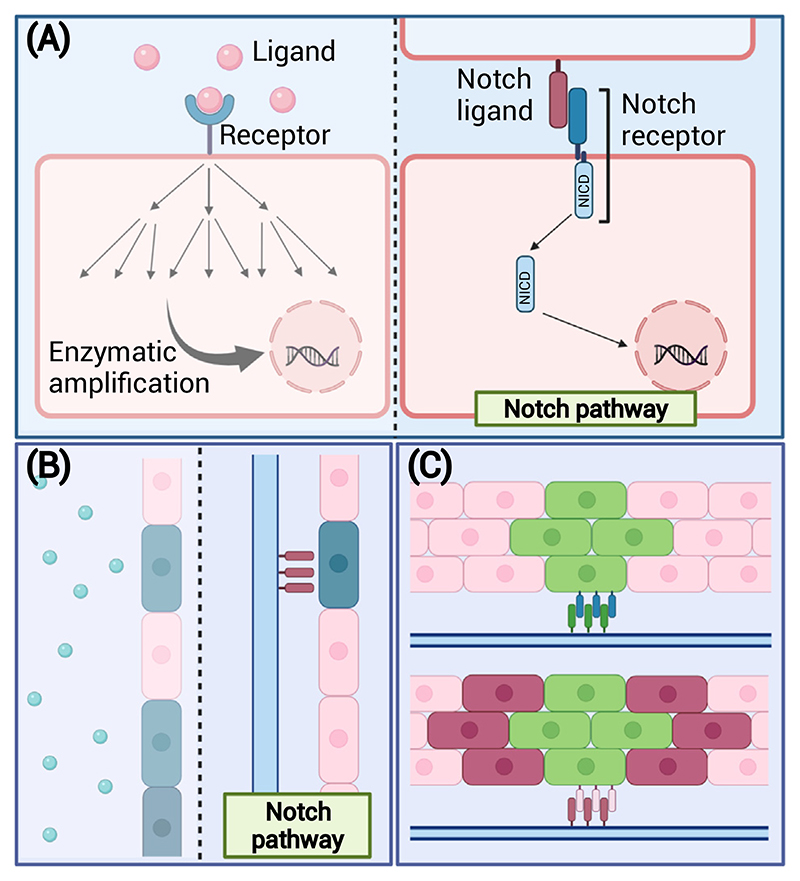
Engineering-friendly properties of Notch signaling. (A) Unlike many signaling pathways, the extent of Notch signaling can be controlled by adjusting the number of the receptor-ligand interactions. (B) Owing to its contact-dependent nature, Notch provides local precision for controlling cell fate. (C) Different cellular responses can be achieved by targeting different Notch receptors/ligands in different tissue contexts by using the same principles. Abbreviation: NICD, Notch intracellular domain.

Notch exhibits exceptional dose-sensitivity. Deletion/dysfunction of a single Notch receptor or ligand allele leads to drastically different biological outcomes. The Notch receptor is a singlepass transmembrane protein, and its activation yields only one active intracellular fragment, the Notch intracellular domain (NICD). In contrast to many other pathways, this process does not rely on further enzymatic amplification (Figure 3A). Instead, the outcome of Notch activation follows stoichiometric interactions between the components of the pathway [34,35]. Notch target genes have been suggested to respond to different levels and dynamics of the NICD [36]. Such properties make the outcome of the pathway sensitive to small changes in the membrane levels of its receptors and ligands [34,36]. Further, the Notch pathway comprises multiple ligands which activate distinct genetic programs in distinct biological contexts as described earlier.

From an engineering point of view, the linear stoichiometry of the Notch NICD and its dosedependent activity and ligand specificity allow Notch signal intensity and its outcome to be controlled by adjusting the local ligand concentration and identity so as to fine-tune cellular responses.

### Notch allows spatial control of cell fate

As illustrated by the examples mentioned in the preceding text, native tissues are composed of multiple cell types that form a hierarchical organization important for tissue function. To achieve this functional hierarchy in engineered tissues local cell fate control is necessary. Native Notch signaling is activated through the interaction between a membrane-bound ligand and the receptor. In a similar fashion, artificial environments require that the ligands are surface-bound before they can activate the pathway [25,37]. In fact, soluble ligands and decoys compete with membrane-bound ligands to inhibit Notch activation [38,39]. Thus, tethering the ligands to materials allows localized Notch activation, either via homogeneously distributed or patterned ligands (Figure 3B). Therefore, Notch signaling can be used to control cell fate with local precision in scaffold environments.

### Notch is a versatile tool in multiple tissue contexts

A single Notch input can propagate across cell populations through lateral induction or lateral inhibition (Figure 2 third tier, and Figure 3C), leading to diverse cell fates. In addition, which of the two signal-induction mechanisms takes place *in vivo* depends on the biological context and on cell-intrinsic properties such as the balance between the membrane levels of receptors and ligands, the dominant ligand type, and post-translational modifications that fine-tune the interactions between distinct receptor-ligand pairs [7]. This means that by targeting different receptors it is possible to control different cellular processes, and that the same engineering principles can be reused in various tissue contexts.

## Activating Notch for tissue patterning: design considerations and potential

Research on modulating Notch has mostly, but not exclusively, focused on inhibition of Notch in cancer therapy (Box 2) [38,39]. For tissue engineering purposes the activation of Notch has been highly desirable and a main point of focus. Traditionally, coculture systems have been used to activate Notch through cell–cell interactions. However, these systems provide poor specificity for Notch signaling and give rise to non-specific cellular responses, and thus may be of little relevance for tissue engineering applications [40]. Early on Varnum-Finney et *al.* found that ligand immobilization can induce Notch receptor activation [41], opening the doors for mimicking native cell-cell interactions by well-defined cell–material interactions. Efforts have since been made to develop Notch-activating platforms, and today Notch-functionalized materials range from tissue-culture plastics [24,25,27,41–46] to 3D matrices [37,47–53] and intricate dynamic systems such as magnetic nanoparticles and photo-reversible Notch-activating gels (Figure 2, fourth tier) [54–57] (Table 1). However, challenges remain for robust control over Notch as well as in its systematic use to control tissue morphogenesis. In the following section we summarize our current understanding of material–mediated Notch activation and strategies to successfully manipulate the outcome.

### Ligand immobilization

Notch activation is not driven by straightforward receptor allostery. Following the receptor–ligand interaction, neighboring cells exert microforces that pull on the Notch receptor to reveal an enzymatic cleavage site [58]. These forces are essential for receptor activation and are thought to be generated by sender cells through endocytosis [59,60] and cytoskeletal interactions [61,62]. This pulling can be recapitulated by immobilized ligands, presumably because of the resistance provided by surface-tethering. However, receptor–ligand interactions are fine-tuned by several other factors, including the orientation and the receptor affinity of the ligands, as well as by ligand–lipid interactions [43,55,63–65] which can be perturbed by ligand modifications introduced for the purpose of immobilization. Multiple approaches for ligand immobilization have been tested to optimize ligand bioactivity while still catering for engineering needs.

Notch ligands are long polypeptides (>500 amino acids) and are thus difficult to modify or immobilize in an orthogonally well-defined fashion through conventional crosslinking methodologies. Shorter ligand-mimicking peptides, such as a 17-amino acid Jag1 DSL domain fragment, have been tested for covalent conjugation to materials [52,66,67]. However, these peptides do not appear to be as potent in inducing Notch as the full-length ligands [67]. Currently, the most commonly and recently used molecules for Notch activation are chimeric or tagged ligands consisting of the extracellular domains of the Notch ligands fused to, for example, antibody fragment crystallizable (Fc) regions [38,44,53,55,68]. Although these ligands can be immobilized directly through physical or chemical adsorption [47,48,56], the tags and chimeric fragments allow their indirect immobilization through high-affinity interactions with secondary docking molecules such as antibodies or protein A/G [27,41,49,50,69–72]. The latter has been shown to facilitate correct ligand orientation and enhance Notch activation potential in comparison to direct methods, especially physical adsorption [37,43,45,46]. Based on an *in vitro* Notch activation assay with luciferase reporter cells, Gerbin and colleagues showed that Dll1 immobilized within collagen hydrogels through anti-IgG induces a dose-dependent increase in the Notch signal, whereas direct physical immobilization of the ligand did not lead to a significant response [37].

### Spatiotemporal control

As in the example of the developing heart given previously, Notch regulates the morphogenesis of different regions of the heart at different stages of development. The spatial distribution of the Notch signal therefore determines the final form of the tissue. Therefore, high spatial precision is necessary to control patterning. Conversely, spatial regulation provides an opportunity to engineer the structure of more complex tissues through Notch modulation. The layering of specific cell types unique for tissue function (e.g., in valvular tissues) and controlled formation of ducts (e.g., in organs such as liver or pancreas) might be improved through spatial and/or temporal control of Notch stimuli. Hence, spatial control of Notch activation is an important design feature for materials for tissue patterning and engineered morphogenesis. Notch also elicits different responses during tissue development as a function of temporal regulation [12,73]. A small number of studies have investigated methods for spatial and/or temporal control of Notch (Figure 2, third tier) [30,40,56,57]. One study utilized printed lines of Dll4 ligands to spatially guide the direction of the endothelial sprouts. Dll4 lines restricted the origin and direction of the endothelial sprouts between the printed ligand lines, and hence may be applied to spatially guide angiogenesis and vascularization of engineered tissue constructs [30]. Another study used removable microparticles to refresh and control ligand dosage in a temporally controlled fashion. Fibrin microparticles coupled to Jag1 were used to maintain human embryonic stem cell-derived cardiomyocytes under prolonged culture by repeated addition of new doses of Jag1, thus eliminating the need to subculture [40]. Both the spatial and the temporal control approaches we have highlighted were efficient in 2D as separate systems, but did not address combinatorial spatial and temporal control or translation to 3D environments. To address combinatorial spatial and temporal control, DeForest and Tirrell developed a hydrogel technology for 3D reversible photo-patterning of proteins within a polyethylene glycol (PEG) hydrogel matrix [56]. By using photocaged sites and additional photo-scissible linkers, the authors were able to both immobilize Notch ligands in the 3D hydrogel at defined sites and remove the ligands on demand with a resolution of a few micrometers [56]. Similarly, Rizwan and colleagues followed a ligand-concealing approach by chemically modifying Jag1 ligands with streptavidin cages using photo-scissible crosslinkers, which allowed the creation of active ligand patterns and on-demand activation on the hydrogel [57]. However, in addition to the on-and-off control described in these examples, Notch signaling *in vivo* is also subject to temporal control through oscillatory patterns of ligand expression. Oscillatory Notch activity has been indicated in processes such as stem cell and progenitor maintenance in skeletal muscle, brain, and pancreas [74–76]. For example, in skeletal muscle progenitors, Dll1-mediated Notch activity drives *Hes1* gene expression, whereas Hes1 protein represses the expression of *Dil1*. This generates a continuous negative feedback loop that leads to oscillations in the expression of *Dil1* and *Hes1* which are coupled to expression of tissue-specific transcription factors that regulate proliferation and differentiation. Inducing sustained high Notch activity versus oscillatory activity leads to different cellular outcomes, such as stem cell quiescence or proliferation in adult skeletal muscle, respectively [15]. Thus, it might be relevant not only to induce Notch signaling but also to consider and control its oscillations when engineering tissue morphogenesis, for example to maintain the stem cell pool. Oscillatory ligand presentation has not yet been investigated in the context of Notch modulation. However, one can speculate that a level of temporal resolution, such as an oscillatory periodicity of 2–3 h, – as seen in myogenic cells in mice [74] – can be attained in the future, for example through approaches similar to the photolithographic patterning examples given. In this scenario, the challenge remains for efficient immobilization and removal of the added/liberated soluble ligands because soluble ligands may interfere with ligand–receptor binding and inhibit Notch activation. Altogether, the current state of the technology offers a starting point for the creation of sophisticated material designs for spatial and temporal control of Notch in tissue-patterning contexts.

### Fine-tuning receptor–ligand interactions

As discussed in the preceding text, receptor activation depends on multiple factors, including force. The magnitude of the pulling forces might be important for ligand discrimination during cell–cell interactions. For example, Jag1 and Dll4 have both been shown to form catch-bonds with Notch1, but they require different magnitudes of force to activate Notch1 [77]. On the other hand, a 9 pN force is sufficient to activate Notch and can override ligand identity [55]. However, the pulling forces in the case of immobilized ligands have not been fully characterized. This raises the question of whether excessive forces might hamper ligand specificity, and also of whether softer substrates might fail to provide sufficient pulling force. Consistently, the evidence suggests that substrate stiffness synergizes with immobilized ligands to fine-tune Notch signaling [78]. To this end, it may be worthwhile to characterize these forces and tune them towards physiological signaling, for example through tension gauge tethers [55,64,79].

Furthermore, the ligands are differentially processed based on their endodomains, thereby leading to different responses through the same Notch receptor [80]. Dll4 and Dll1 have been suggested to be differently internalized by the sender cells, and thus may pull on the receptors as single species or in bulk, leading to either a sustained signal or a single large pulse – which result in different transcriptional responses through the same Notch receptor [80]. Thus, it may be relevant to develop tools that can fine-tune receptor dosing in a similar fashion. In this context, ligand-bearing magnetoplasmonic gold nanoparticles (MPNs) are a good inspiration [55]. By using a combination of magnetic tweezers and MPNs, Seo and colleagues were able to move receptors along the cell membrane to control receptor clustering with a 1 pN force. However, a 9 pN force was required for the MPNs to successfully activate Notch [55]. By extension, the incorporation of artificially induced microforces and controlled clustering may increase the specificity of Notch-activating material systems, although their incorporation into 3D systems might prove challenging.

## Remaining challenges and considerations

When steering Notch signaling through the use of functionalized materials such as ligand-functionalized surfaces, it is important to consider the physical properties of the engineered microenvironment. These include factors that affect Notch signaling, such as environmental mechanics and stiffness, and that can either be utilized for optimum control over Notch signaling-induced cell fate or to counteract its effects. Studies on Notch-functionalized hydrogels reported that matrix stiffness affects intrinsic Notch signaling, and that the interplay between Notch and the spatial distribution of microforces deriving from matrix stiffness regulates cell fate decisions [70]. Furthermore, a recent experimentally informed mathematical model on the arterial wall suggests that hemodynamic strain affects the sender–receiver behavior of VSMCs through Jag1-Notch3 interactions, and thereby the propagation of the Notch signal during arterial growth and remodeling [22]. These findings illustrate the importance of the intrinsic properties of the materials as well as the involvement of macro-level tissue mechanics when using Notch as a tool for tissue engineering and patterning. Another challenge is that tissues with a complex architecture, which are built via multiple patterning processes, rely on the sequential delivery of multiple different Notch ligands. Examples include the stage-specific activity of different Notch ligands in the development of the ventricular chambers, valves, and the organ of Corti [7,17,18]. Such selective ligand activity *in vivo* is mediated, for example, by post-translational modifiers such as fringe glycosyltransferases (Manic-, Lunatic-, and Radical-Fringe) [7] that fine-tune receptor–ligand interactions. Based on this knowledge, tools that can facilitate the sequential and specific delivery of the ligands are needed, although challenges remain in achieving sequential ligand activity in engineered *in vitro* systems that recapitulate the processes taking place *in vivo*.

## Concluding remarks

For a long time Notch was considered to be a difficult therapeutic target. With the advent of immobilized ligands and materials technologies, Notch activation has extended to tissue engineering applications. Proof-of-concept tools that can help to control tissue patterning through Notch are available. However, some challenges and considerations remain for their use in the generation of complex tissues (see Outstanding questions).

First, our knowledge of the biology of Notch signaling and the development of technologies to utilize it go hand in hand. Although advanced materials for Notch modulation have been developed over the years, very few attempts have been made to use them to control tissue patterning. We believe that this is partly due to insufficient mechanistic understanding of the molecular events taking place during patterning. Although a large amount of biological data on the role of Notch in organ development has emerged from gain/loss-of-function models, the involvement of different Notch components and the exact sequences of events they mediate are still insufficiently understood. As illustrated by the examples we have given, paralogs of Notch receptors and ligands may be involved in different developmental timeframes and control different cell fate decisions. Thus, understanding the temporal activity, dosing, and redundancy of different Notch ligands/receptors and their roles in the patterning of different tissues via time-resolved experiments will aid in the design of material-based approaches for steering tissue patterning *in vitro* or *in vivo.*

Second, the engineering systems are only as good as their controllability. Based on the examples we have introduced, Dll and Jag activities are often connected to lateral inhibition and lateral induction, respectively. Thus, we can speculate that lateral inhibition and induction events might be potentiated in other tissue contexts that remain to be uncovered. Therefore, patterned ligands may not only be used to control cell fate decisions in predefined areas but could also act as initial signals to steer cell-intrinsic feedback loops to achieve self-assembly of cells. *In vivo*, lateral patterning arises from initially small stochastic molecular events. Ligand-functionalized materials may provide this initial stimulus with local precision and trigger self-propagation of the Notch signal leading to patterning events (e.g., patterning of *in situ* engineered vessels). However, predicting the long-range outcome of such stimuli will be necessary in controlling complex tissue patterning. To this end, we believe that synthetic biological models and predictive tools such as organoids, biomimetic microtissues, and computational models will contribute by providing insight into how a Notch signal induced by extrinsic factors propagates through cell-intrinsic feedback mechanisms in concert with other environmental cues. Some computational models have already been developed for Notch signaling in tip versus stalk cell selection via lateral inhibition during angiogenesis, and VSMC phenotypic regulation via lateral induction during vascular remodeling. Ideally, insights from such models will translate to future design strategies for tuning the form, localization, and duration of extrinsic Notch signals for engineering tissue pattern formation, such as in directed vascularization of 3D tissue grafts or engineering of arterial walls.

Taken together, despite current challenges, the role of Notch in tissue morphogenesis and the engineering-friendly nature of Notch make it a potential tool for steering tissue patterning in multiple tissue contexts. Modulation of Notch has not only contributed to stem cell control and expansion but has also provided handles for use in regenerative contexts such as wound healing and vascularization, as well as in inspired engineering toolboxes and material design concepts for engineering complex tissue morphogenesis. As such, dedicated analysis and targeting of Notch signaling through interdisciplinary efforts will contribute to the generation of morphologically and functionally accurate tissue constructs.

## Figures and Tables

**Table 1:** Overview of studies of Notch ligand-functionalized (bio)materials and their respective target tissues

Ligand	Cell/tissue type	Refs	Notes
Dll1	Muscle	[41,78]	
	Immune/blood	[24,27,47,53,54,69,85]	
	Dental	[42]	
	Cardiac/stem cells	[37]	
	-	[55]	
Dll4	Hepatic	[70]	
	Reporter cells	[86]	
	Immune/blood	[48,87]	
	Cancer/HeLa cells	[68]	
	Endothelial	[30]	
Delta^a^	Reporter cells	[56]	
Jag1	Immune/blood	[43,46]	
	Epithelial	[44], [45]^b^, [66]^b^, [88]^b^	Peptide^b^
	Dental	[25,42,89–91]	
	Muscle	[78]	
	Cardiac	[40]^b^, [51]^b^	
	Reporter cells, stem cells	[49], [52]^b^, [67]^b^, [72]^b^	Peptide^b^
	Bone	[71]	
	Hepatic	[57]	

a Ligand number was not specified in this study.

b Peptide was included in the study.

## Box 1 The Notch signaling pathway

Notch signaling is a contact-dependent pathway that is evolutionarily conserved in metazoans. In humans, Notch signaling is activated through the interactions of the Notch ligands Jagged (Jag) 1,2 and Delta-like ligands (Dll)-1,3,4 with Notch receptors (Notch 1,2,3,4) that are present on the membranes of a ligand-presenting (sender) and a receptor-presenting (receiver) cell (Figure I). Each Notch receptor is a heterodimer of two subunits – a transmembrane/intracellular domain and a Notch extracellular domain (NECD). Upon receptor-ligand interaction, the sender cell internalizes the ligand together with the NECD [81]. This creates a mechanical pull on the extracellular domain of the receptor to unfold the negative regulatory region (NRR) and reveal the S2 proteolytic cleavage site for a disintegrin and metalloprotease (ADAM) metalloproteinases. S2 cleavage is then followed by S3 cleavage mediated by y-secretase that releases the active intracellular domain (NICD), which then translocates to the nucleus and interacts with CSL transcription factors to drive the transcription of Notch target genes [63]. Notch regulates important cell fate decisions such as migration, proliferation, differentiation, and apoptosis [6]. In addition to controlling cell-intrinsic programs, the Notch signal can be processed in neighboring cells through positive and negative feedback loops that induce or repress ligand expression in the signal-receiving cell. These processes are called lateral induction and lateral inhibition, respectively. Which of these two processes becomes prominent is determined by the cellular context. Lateral induction promotes the same cell fate in the neighboring cells, whereas lateral inhibition leads to adoption of differential cell fates. These lateral events give rise to patterning of cell populations and contribute to their morphogenesis as functional compartments [7,81]. Notch is involved in development from somitogenesisto postnatal stages, as well as in homeostasis, regeneration, and cancer in many adult tissues [6].

## Box 2 Small drugs and other molecules for Notch modulation

Given the involvement of Notch in both development and disease, targeting Notch for a plethora of therapeutic purposes has been researched extensively [38]. These efforts have yielded multiple active molecules such as small-molecule drugs (e.g., Y-secretase inhibitors), antibodies, peptides, and microRNAs that target ligand-receptor interactions, receptor processing, or the expression of Notch pathway components [38,39,82–84]. Most were developed as inhibitors of the pathway, although some could be used as activators. Although many of these molecules are in clinical trials, none are currently in clinical use, principally because of adverse systemic effects (reviewed in [38,39]). Thus, these molecules have not been extensively studied for tissue-engineering applications. However, in the future they might become valuable tools for modulating cell fate provided that they can be integrated into engineering systems. In particular, the intriguing specificity of receptor-activating or -blocking antibodies poses the question whether they can be used in material-mediated strategies for receptor-specific modulation of Notch.

## Highlights

Notch signaling drives tissue patterning and morphogenesisduring development and in the adult. Thus, targeting Notch can helpto engineertissueswith defined form and function.

Mimicking Notch-mediated patterning will require high spatiotemporal resolution for recapitulating *in vivo* patterning events.

Targeting Notch signaling through materials functionalized with immobilized ligands is an engineering-friendly tool for controlling cell fate decisions with local precision and dose control.

Important challenges remain in transforming the available concepts and tools into effective tissue-patterning strategies for regenerative medicine.

## Outstanding questions

To what extent can we predict the correct localization and timing of Notch activation during tissue morphogenesis? Computational models have been developed to elucidate the complex feedback and feedforward loops involved in Notch signaling. How can we verify and utilize computational models, informed by quantitative experimental data, to help us steer engineered Notch-driven tissue morphogenesis?

Can we use a transient and local Notch signal to trigger cell populations into a self-sustaining state and induce morphogenesis? Thus, will a single, initial localized signal mediated by immobilized ligands be sufficient to trigger a population-wide signaling cascade through lateral induction and inhibition to guide self-organization?

How can we sequentially deliver different Notch-controlling molecules for consecutive physiological-like patterning events with local and temporal specificity? The patterning of many organs depends on sequential activation of Notch ligands and receptors. To address this, can we integrate multiple different ligands into the current spatiotemporal patterning technologies and systematically control their activity in space and time to control tissue patterning?

Can we combine the cell-instructive power of Notch signaling with other engineering techniques, such as bioprinting, for better control over tissue generation? By following a hybrid patterning approach, can we enhance both the architecture and the functionality of a tissue?

## Key figure

Notch signaling modulation for creating tissues with physiological form and function

## Glossary

### Abbreviations

A disintegrin and metalloprotease (ADAM): enzymes of this class cleave the transmembrane domain of the Notch receptor to release the Notch extracellular domain before Y-secretase-mediated cleavage of the Notch intracellular domain.
Delta-like ligands 1,3,4 (Dll1/3/4): Notch ligands; lateral inhibition is often associated with Dlls because they generally downregulate their activation in the signal-receiver cell.
DSL domain: a protein domain (named from Delta/Serrate/lag-2) that is present in Jagged1. This domain has been suggested to be crucial for ligand-mediated activation of Notch.
Fragment crystallizable (Fc) region: the tail region of antibodies. Protein A/G has high affinity for Fc. Thus, engineered peptides containing this fragment can be indirectly immobilized on surfaces precoated with protein A/G.
Intestinal stem cells (ISCs): located in the crypt base, ISCs renew continuously, while some of them differentiate to functional cells.
Jagged 1 and 2 (Jag1/2): Notch ligands that are often associated with lateral induction because Jagged is generally upregulated by Notch signaling. They are longer than delta-like family ligands.
Lateral induction: the process of sequential Notch signaling in which the receiver cell is induced to express the same ligands as the sending cell, and can thus propagate the signal further to the next receiver cell.
Lateral inhibition: the process of differentially inducing cell fate in which the receiver cell downregulates the same Notch ligands as the sending cell had presented, thus maintaining the difference in cellular target gene induction and thus the differential cell fate of neighboring cells.
Magnetoplasmonic nanoparticles (MPNs): nanoparticles that can be manipulated in space through magnetism.
Negative regulatory region (NRR): this region of the receptor is unfolded upon pulling on the receptor by its ligand. This exposes a cleavage site, and ADAM can then cleave the receptor.
Notch extracellular domain (NECD): after Notch cleavage by ADAM, this fragment is detached from the signal-receiving cell. It is thought to be internalized together with the ligand by the signal-sender cell.
Notch intracellulardomain (NICD): the active fragment of Notch receptor generated by y-secretase cleavage; the NICD translocates to the nucleus where it modulates gene expression.
Polyethylene glycol (PEG): a polymer that can form hydrogels and is commonly used in scaffold fabrication.
Vascular endothelial growth factor receptors (VEGFRs): a group of cellular receptors involved in vascular development and branching.
Vascular smooth muscle cells (VSMCs): cells in arterial and venous blood vessels that regulate arterial structure and contractility.
